# Antioxidant and DNA-Protective Potentials, Main Phenolic Compounds, and Microscopic Features of *Koelreuteria paniculata* Aerial Parts

**DOI:** 10.3390/antiox11061154

**Published:** 2022-06-13

**Authors:** Tsvetelina Andonova, Yordan Muhovski, Radka Vrancheva, Ilya Slavov, Elena Apostolova, Samir Naimov, Atanas Pavlov, Ivanka Dimitrova-Dyulgerova

**Affiliations:** 1 Department of Botany and Biological Education, Faculty of Biology, University of Plovdiv “Paisii Hilendarski”, 4000 Plovdiv, Bulgaria; ts_andonova@uni-plovdiv.bg (T.A.); ivadim@uni-plovdiv.bg (I.D.-D.); 2 Life Sciences Department, Walloon Agricultural Research Centre, 5030 Gembloux, Belgium; 3 Department of Analytical Chemistry and Physical Chemistry, Technological Faculty, University of Food Technologies, 4002 Plovdiv, Bulgaria; r_vrancheva@uft-plovdiv.bg (R.V.); a_pavlov@uft-plovdiv.bg (A.P.); 4 Department of Biology, Faculty of Pharmacy, Medical University of Varna, 9000 Varna, Bulgaria; ijelev80@abv.bg; 5 Department of Plant Physiology and Molecular Biology, Faculty of Biology, University of Plovdiv “Paisii Hilendarski”, 4000 Plovdiv, Bulgaria; eapostolova@uni-plovdiv.bg (E.A.); naimov0@uni-plovdiv.bg (S.N.); 6 Laboratory of Cell Biosystems, Institute of Microbiology, Bulgarian Academy of Sciences, 139 Ruski Blvd., 4000 Plovdiv, Bulgaria

**Keywords:** antioxidant activity, DNA nicking protection, ethanol extracts, flavonoids, HPLC analysis, *Koelreuteria paniculata*, phenolic acids, microscopic diagnostic features

## Abstract

Interest in plant extracts as a natural source of antioxidants has grown significantly in recent years. The tree species *Koelreuteria paniculata* deserves attention due to its wide distribution, good adaptability, and growth to the degree of invasiveness in a number of European countries. The purpose of the present study was to analyze flavonoids and phenolic acids of the ethanol extracts from aerial parts of *K. paniculata* and to screen their antioxidant and DNA-protective activity. HPLC profiling revealed the presence of five flavonoids, with rutin (4.23 mg/g DW), hesperidin (2.97 mg/g DW), and quercetin (2.66 mg/g DW) as the major ones in the leaves, and (−)-epicatechin (2.69 mg/g DW) in the flower buds. Among the nine phenolic acids identified, rosmarinic, *p*-coumaric, salicylic, vanillic, and gallic acids were the best represented. All the extracts tested showed *in vitro* antioxidant activity that was determined by DPPH, ABTS, FRAP, and CUPRAC assays. The highest activity was recorded in the flower parts (in the range from 1133 to 4308 mmol TE/g DW). The DNA-protective capacity of the flower and stem bark extracts from the *in vitro* nicking assay performed, as well as the main diagnostic microscopic features of the plant substances, are given for the first time. According to the results obtained, the aerial parts of *K. paniculata* could be valuable sources of natural antioxidants.

## 1. Introduction

The damaging effects of reactive oxygen species (ROS) on cellular structures can cause various diseases [1,2,3]. A wide range of ROS is formed *in vivo* in the human body and other living organisms. Some ROS of less reactivity play an important physiological *in vivo* role, while those of higher reactivity cause oxidative damage to biomolecules. Oxidative damages to DNA caused by ROS lead to the formation of various mutagenic end products that, in turn, cause the emergence and progression of many human diseases [3].

Most antioxidants are natural plant compounds that can slow or stop the occurrence of degenerative reactions in the body [1,3,4,5], and they may be a promising source for the prevention or treatment of free radical-generated diseases such as atherosclerosis, hypertension, diabetes, cancer, Parkinson’s, Alzheimer’s, etc. [6]. By participating in DNA damage protection, they can prevent or modulate the process of carcinogenesis [1].

In many studies, flavonoids are indicated as the phytochemicals with the highest antioxidant activity [7,8]. They are a large group of phenolic substances, secondary metabolites that are found in different parts of all vascular plants, which act as growth regulators and participate in the protection against oxidative stress by controlling the accumulation of ROS [4,7,8]. The prevention and treatment of many diseases that involve flavonoids are well known for the mechanisms of making free radicals harmless and inhibiting the factors that cause disease [4,7]. Their effectiveness has been defined, and they are considered agents that are reliable antioxidant, anticancer, antibacterial, antiparasitic, cardioprotective, hepatoprotective, neuroprotective, immunomodulatory, antidiabetic, anti-inflammatory, and even potential antiviral agents [2,4,8,9]. In recent years, phenolic acids have been the focus of more and more clinical research due to a number of their health-protective effects [10]. Epidemiological data show that phenolic acids reduce the risk of many diseases associated with oxidative stress, namely cancer, diabetes, and cardiovascular disease [11]. Plant extracts are a natural source of flavonoids and phenolic acids, and that is the reason for the increasing interest in them. 

Our scientific interest was focused on the tree species *Koelreuteria paniculata* Laxm (Sapindaceae), which naturally grows in North China, Japan, and Korea, and it is naturalized as an ornamental park tree in Europe. Subsequently, it has been declared an invasive or potentially invasive species in a number of European countries, including Bulgaria [12]. Moreover, its good adaptability, vitality, and growth can be useful in the search for new, accessible, and cheap sources of pharmaceutically active components.

The scientific literature review on phenolic compounds of *K. paniculata* shows mainly analyses of total polyphenols and flavonoids by the application of quantitative spectrophotometric methods [13,14,15,16,17]. Some phenolic components in leaf extracts have been reported by Lin et al., Mostafa et al., and Mahmoud et al. [18,19,20], including hyperin, catechin, galloylepicatechin, isorhamnetin, quercitrin, quercetin-3′-O-*β*-D-arabinopyranoside, 5-methoxy luteolin, gallic acid and its derivatives, etc. *K. paniculata* leaf fractions with proven antibacterial and antifungal activities show the content of pyrogallol, gallic acid, isobutyl gallate, benzoic acid, phenylacetic acid, and other secondary metabolites [21]. Chromatographic analyses of extracts from branches and leaves found the content of a large number of active substances, including some phenolic compounds—pyrogallol, ethyl gallate, and methyl ester of benzoic acid [22]. Gallic acid, kaempferol, luteolin, hyperoside, and five more flavonol glycosides of quercetin and kaempferol from the flowers were also isolated [23]. Paniculatonoids A and B [24], flavonoids, and cycloartane glycosides [25] were found in seeds. The comparative analysis of total flavonoids from different plant parts found the highest content in fruits, and it increases during the ripening process, followed by leaves and branches [17].

The good antioxidant potential and DNA-protective effect of extracts and their fractions of *K. paniculata*, which correspond to the quantitative content of total polyphenols and flavonoids in them, have been shown by other authors [13,14,15]. Based on the proven antioxidant activity, leaf extracts have been identified as a potential inhibitor of lipid peroxidation and 4-nitroquinoline-1-oxide (4-NQO)-induced DNA damage and good effectiveness against free radicals and H_2_O_2_-induced damage to DNA [13,14,16].

Our previous studies have supplemented the knowledge about the essential oil content and chemical composition of ethanol extracts from the aerial parts of the species [26,27]. The rich composition of terpenes, terpenoids, and phenylpropanoids played a determining role in the antibacterial and antitumor activities proven by us.

Considering the above and the fact that there is incomplete information that concerns the composition of phenolic compounds and the potential of *K. paniculata* for free radical scavenging, such data are missing for the species that grow in Bulgaria. As a result, the aim of the present study was to analyze extracts from the aerial parts of *K. paniculata* for the presence of the main phenolic acids and flavonoids and the ability of those extracts to protect against oxidative damage. In addition to this goal, the task was set to analyze the main microscopic diagnostic features of the plant substances.

## 2. Material and Methods

### 2.1. Chemicals and Reagents

*Reagents used for HPLC analysis and antioxidant activities*. The following reagents were purchased from Sigma-Aldrich Chemie GmbH (Steinheim am Albuch, Germany): potassium persulfate, sodium acetate anhydrous, DPPH (2,2-diphenyl-1-picrylhydrazyl), ABTS (2,2′-azino-bis(3-(6-hydroxy-2,5,7,8-tetramethylchroman-2-carboxylic acid), TPTZ (2,4,6-tri(2-pyridyl)1,3,5-triazine), iron (III) chloride, neocuproine, copper (II) chloride, ammonium acetate, rutin, hesperidin, kaempherol, (+)-catechin, (−)-epicatechin, protocatehuic acid, gallic acid, chlorogenic acid, vanillic acid, caffeic acid, syringic acid, *p*-coumaric acid, ferulic acid, salicylic acid, rosmarinic acid, and HPLC-grade solvents (acetonitrile, methanol, ethanol, and acetic acid).

*Reagents used for DNA nicking protection assay*. Trolox(6-hydroxy-2,5,7,8-tetramethylchromane-2-carboxylic)—Sigma-Aldrich, cat No. 238813; Potassium phosphate dibasic—Sigma-Aldrich, cat No. P3786; di-Potassium hydrogen phosphate—Merck (Darmstadt, Germany), cat No. 1051015000; Iron(II) sulfate heptahydrate—Merck, cat No. F7002; Hydrogen peroxide solution—Sigma, cat No. H1009; TBE buffer—Duchefa (Haarlem, The Netherlands), cat No. T1507; Agarose SPI—Duchefa, Cat No. A1203; 96% Ph. Eur., extra pure, Karl Roth, Germany); Whatman filter paper No. 1 (Sigma-Aldrich, Steinheim am Albuch, Germany).

### 2.2. Plant Material

The plant parts of *Koelreuteria paniculata* (stem bark, leaves, flower, and flower buds) were collected in May–July 2020 from Bulgaria (Plovdiv city, 42°8′9.9492″N, 24°44′31.8048″E). They were systematically identified at the Department of Botany, Faculty of Biology, University of Plovdiv “P. Hilendarski”. The voucher specimen of the species was deposited (No. 060436) in a herbarium (SOA) at the Agricultural University of Plovdiv, Bulgaria. Fresh plant material was used for DNA nicking protection assay, and for other analyses, plant samples were dried at room temperature, ground, and stored in glass vials before use.

### 2.3. Preparation of the Plant Extracts

One gram of dried plant samples (hydromodule 1:10) was extracted three times with 70% water–ethanol (*v*/*v*), at 70 °C in a water bath, heated under reflux for 15 min. The residue of the plant material was removed through filter paper filtration, and the combined ethanol extracts were used for HPLC analysis of the phenolic profile and antioxidant activity.

The fresh and crushed plant parts were extracted with 96% ethanol in the dark for 10 days. A vacuum evaporator (Buchi, Rotavapor R-300) was used at 50 °C and 97 mbar to concentrate the resulting extract after prefiltration through a Whatman filter paper No. 1. The dry extracts were collected in a vial and stored at 4 °C, in the dark, for DNA nicking protection assay.

### 2.4. Microscopic Analysis

The ground plant samples of *K. paniculata* were subjected to microscopic analysis using *chloral hydrate solution* to establish the main diagnostic pharmacognostic features. The samples were sieved through a pharmacopoeial sieve (aperture size—0.4 mm) before microscopy. For this purpose, the Magnum T Trinocular microscope CETI (Medline Scientific, Oxfordshire, UK) was used at the Department of Botany, the University of Plovdiv “P. Hilendarski”. Light micrographs were taken with a photodocumentation system Si 5000 5 Mpx (Medline Scientific, Oxfordshire, UK), coupled with the microscope.

### 2.5. HPLC Analysis of Flavonoids and Phenolic Acids

HPLC analysis was performed according to Krasteva 2022 [28] using Waters 1525 (Binary HPLC pump), UV-VIS Waters 2487 (Dual λ Absorbance Detector), and a SUPELCO Analytical Discovery HS C18 column (25 cm × 4.6 mm, 5 µm). The injected sample was 20 µL. Elution of the compound was performed by a gradient of 1% acetic acid in water (Mobile phase A) and methanol (Mobile phase B) at a speed of 1 mL per minute. The elution program was: 1–36 min 90% A and 10% B, 36–37 min 78% A and 22% B, 37–47 min—70% A and 30% B, 47–58 min 60% A and 40% B, 58–59 min 54% A and 46% B, 59–71 min 40% A and 60% B, 71–72 min—20% A and 80% B, and 72–75 min—90% A and 10% B. The detection was carried out at λ = 280 nm /gallic acid, protocatechuic acid, (+)-catechin, vanillic acid, syringic acid, (−)-epicatechin, *p*-coumaric acid, salicylic acid, hesperidin/ and λ = 360 nm /chlorogenic acid, caffeic acid, ferulic acid, rutin, rosmarinic acid, quercetin, and kaempferol/. Representative HPLC chromatograms of the flavonoids and phenolic acids of leaf extracts and the corresponding standards have been added as Supplementary Materials (Figures S1 and S2).

### 2.6. Antioxidant Activity Analyzes

#### 2.6.1. DPPH^•^ Scavenging Assay

The ability of extracts to scavenge 2,2-diphenil-1-picrylhydrazyl (DPPH) radical was determined by the method described by Kivrak et al. and Ivanov et al. [29,30]. A freshly prepared 0.1 mmol solution of DPPH in methanol (2.85 mL) was mixed with a 0.15 mL sample. The light absorption was measured against methanol at 517 nm after 15 min incubation at 37 °C in darkness.

#### 2.6.2. ABTS^•+^ Scavenging Assay

The radical scavenging activity of the extracts against 2,2′-azino-bis (3-ethylbenzothiazoline-6-sulfonic acid) (ABTS^•+^) was estimated according to Thaipong et al. and Ivanov et al. [30,31]. ABTS radical cation (ABTS^•+^) was generated by mixing aliquot parts of 7.0 mmol 2,2′azinobis (3)-ethylbenzthiazoline-6-sulfonic acid (ABTS, Sigma) in distilled water and 2.45 mmol potassium persulfate (Merck) in distilled water. The reaction was performed for 16 h at room temperature in darkness. Before analyses, the generated ABTS**^+^** solution was diluted with methanol in order to obtain the final absorbance of the working solution of about 1.0 ÷ 1.1 at 734 nm. For the assay, 2.85 mL of this ABTS**^+^** solution was mixed with a 0.15 mL sample. After incubation for 15 min at 37 °C in darkness, the absorbance was measured at 734 nm against methanol. 

#### 2.6.3. Ferric Reducing Antioxidant Power (FRAP) Assay

The FRAP assay was carried out according to the procedure of Benzie and Strain, and Ivanov et al. [30,32]. The FRAP reagent was freshly prepared before analysis by mixing 10 parts 0.3 M acetate buffer (pH 3.6), 1 part 10 mmol 2,4,6-tripyridyl-*s*-triazine (TPTZ, Fluka, Buchs, Switzerland) in 40 mmol HCl (Merck), and 1 part 20 mmol FeCl_3_.6H_2_O (Merck) in distilled water. The reaction started by mixing 3.0 mL FRAP reagent with 0.1 mL of the investigated extract. The reaction time was 10 min at 37 °C in darkness, and the absorbance of the sample was recorded at 593 nm against a blank sample that contained 70% ethanol instead of extract.

#### 2.6.4. Cupric Reducing Antioxidant Capacity (CUPRAC) Assay

The CUPRAC assay was carried out according to the procedure of Apak et al. and Ivanov et al. [30,33]. One mL of 10 mmol CuCl_2_ solution was mixed with 1 mL of 7.5 mmol neocuproine (Sigma) in methanol, 1.0 mL 0.1 M ammonium acetate buffer (pH 7.0), 0.1 mL of analyzed extract, and 1.0 mL distilled water. The absorbance of the sample against a reagent blank was measured at 450 nm after incubation at 50 °C in darkness for 20 min.

The antioxidant activity determined by DPPH, ABTS, FRAP, and CUPRAC assays was expressed as mmol Trolox equivalents (TE) per g dry weight (DW) by using a calibration curve built in the range of 0.05–0.5 mmol Trolox (6-hydroxy-2,5,7,8-tetramethylchroman-2-carboxylic acid, Fluka), dissolved in methanol (Merck).

### 2.7. DNA Nicking Protection Assay

The assay was performed using supercoiled pUC19 plasmid as described by Rajiv et al. [34]. Briefly, 500 ng of supercoiled plasmid DNA purified from E. coli strain Neb10 was mixed with Fenton’s reagent (41.5 mmol pH 7.4 phosphate buffer, 0.2 mmol FeSO4, 980 mmol H_2_O_2_), and 4.5 µL from serial dilutions of the plant extracts tested. The concentrations of the extract added per reaction were: 0.6, 1.2, 2.5, 5.0, and 10.0 μg/mL. The same volume of different concentrations (25, 50, and 100 μg/mL) of 6-Hydroxy-2,5,7,8-tetramethylchromane-2-carboxylic acid (Trolox, Sigma) and water were used as a positive and negative control. After 30 min of incubation at 37 °C, the mixture was subjected to 1.5% agarose gel electrophoresis in 0.5× TBE buffer at 50 V for 2 h. The degree of DNA protection was analyzed using the Gel Doc™ EZ Imaging system (Bio-Rad, Hercules, CA, USA). Relative quantification of band intensity was conducted using Image Lab Software (Biorad).

### 2.8. Statistical Analysis

The samples were analyzed in triplicate, and the results were expressed as mean ± standard deviation (SD). One-way analysis of variance (ANOVA) followed by a post hoc Tukey HSD (Honestly Significant Difference) test (online web calculator Astatsa, [35]) was performed to determine significant intergroup differences at a 99% confidence level (*p* < 0.01).

## 3. Results

### 3.1. HPLC Analysis of Phenolic Compounds in Koelreuteria paniculata Extracts

The results obtained by HPLC analysis for the content of phenolic components (flavonoids and phenolic acids) of *K. paniculata* extracts are presented in Table 1. The measured quantitative values of the plant components identified are expressed in mg/g Dry Weight (DW). Fourteen phenolic compounds were identified in the aerial part extracts of *K. paniculata:* five flavonoids (the aglycones quercetin, (+)-catechin, and (−)-epicatechin, and the glycosides rutin and hesperidin) and nine phenolic acids. 

#### 3.1.1. Flavonoid Content

Among the plant parts investigated, the leaves had the highest content of rutin (4.23 mg/g DW), followed by hesperidin and quercetin (2.97 mg/g DW and 2.66 mg/g DW, respectively), as can be seen in Table 1. Flower buds of the species also contained a high concentration of aglycone (−)-epicatechin (2.69 mg/g DW), while quercetin, rutin, and hesperidin showed 11–14 times lower concentrations. In well-developed flowers, the content of (−)-epicatechin decreased 4.5 times. The amount of (−)-epicatechin was the highest in the bark compared with the other flavonoids identified, but in comparison with the flower buds, it was about 3 times lower. Hesperidin was missing in stem bark parts. In our study, the aglycone kaempferol was not detected in any of the samples evaluated, whereas (+)-catechin was identified only in the stem bark of *K. paniculata*.

#### 3.1.2. Content of Phenolic Acids

The different plant parts of *K. paniculata* were screened for 10 phenolic acids (Table 1). In the leaf extract, nine phenolic acids were found, of which rosmarinic acid was in the highest content (10.34 mg/g DW), followed by gallic and vanillic acids. In the flower and flower bud extracts, *p*-coumaric acid (6.97 mg/g DW; 4.97 mg/g DW) and rosmarinic acid (3.00 mg/g DW; 2.62 mg/g DW) were predominant, followed by salicylic acid and protocatechuic acid. The other phenolic acids (vanillic, caffeic, syringic, ferulic acid), in both generative parts, were less represented (in the range between 0.24 mg/g DW and 0.09 mg/g DW). In the bark extract, seven phenolic acids were found in significantly lower content (from 0.01 mg/g DW for protocatechuic acid to 0.22 mg/g DW for rosmarinic acid). Ferulic acid was missing only in this plant part. Gallic acid was only found in leaves, and there was no chlorogenic acid in any of the samples tested. From the phenolic acids studied, those of the highest content (over 1.0 mg/g DW), in descending order, are rosmarinic > *p*-coumaric > salicylic > vanillic > gallic.

### 3.2. Antioxidant and DNA Protective Capacity

DPPH, ABTS, CUPRAC, and FRAP assays were used for the estimation of *in vitro* antioxidant potential of *K. paniculata* extracts (Table 2). All tested ethanol extracts of *K. paniculata* demonstrated the ability to scavenge DPPH radicals and ABTS radical cation, and the highest values were estimated for flower extracts, followed by flower buds extract and leaf extract. The highest reduction ability, determined by FRAP and CUPRAC assays, was also recorded for the flower extract. The most pronounced antioxidant capacity was shown by the flower extracts of *K. paniculata* in the four methods used (3–7 times higher than the lowest values for each method), followed by those of the flower buds, leaves, and stem bark. The arrangement differed only in the CUPRAC assay, where the bark extract followed the flower extract. In most samples, the ferric-reducing antioxidant power was best expressed among the applied methods. The high antioxidant activity, evaluated in the samples analyzed, can be related to the high amounts of phenolic compounds established.

**Figure 1:**
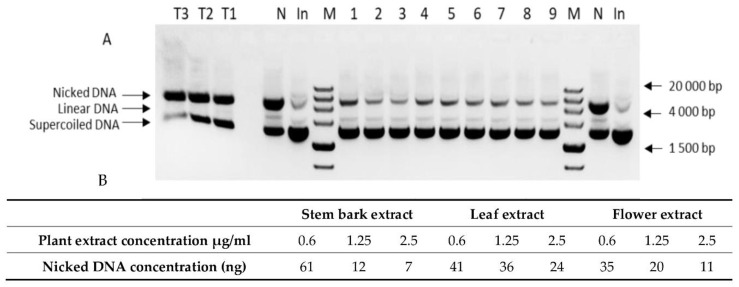
DNA nicking protection assay with (**A**) 1.5% agarose gel electrophoresis, and (**B**) relative concentration of necked plasmid DNA. 1–3—*K. paniculata* stem bark extract at concentrations of 0.6, 1.25, and 2.5 μg/ml; 4–6—*K. paniculata* leaf extract at concentrations of 0.6, 1.25, and 2.5 μg/mL; 7–9—*K. paniculata* flower extract at concentrations of 0.6, 1.25, and 2.5 μg/mL. T1—Trolox 100 μg/mL; T2—Trolox 50 μg/mL; T3—Trolox 25 μg/mL; N—negative control; In—pUC19 input; M—Zip Ruler 2 (Thermo Scientific, SM1373, Waltham, MA, USA).

The DNA-protective capacity of extracts was tested using *in vitro* nicking assay. Complete protection from oxidative DNA damage was found when leaf, flower, and bark extracts were applied in concentrations from 2.5 to 10 μg/mL (data not shown). In order to demonstrate the DNA nicking protection activity of plant extracts, the amounts used for assays were titrated down to 0.6 μg/mL. As shown in Figure 1, at lower plant extract concentrations (0.6 μg/mL, 1.25 μg/mL, and 2.5 μg/mL), the best protective effect was found when bark extracts were used, followed by flower and leaf extracts. In the leaf extracts tested, no increase in the intensity of bands corresponding to the nicked DNA (lines 4–6) was observed. The comparison of nicked DNA band intensity when the flower (lines 7–9) and bark (lines 1–3) extracts are applied as protective antioxidants shows a clear increase in intensity in correlation with the extract concentration. The relative quantification of band intensity showed the best correlation between extract concentration and DNA protective effect when bark extracts are used (lines 1–3). The intensity of nicked DNA bands on line 1 (0.6 μg/mL bark extract) was fivefold higher than the intensity of line 2 (1.25 μg/mL bark extract) and eightfold higher than the intensity of line 3 (2.5 μg/mL bark extract). A similar pattern was observed when flower extract was tested (lines 7–9). A DNA test was not performed on the flower buds because of the fact that they showed antioxidant activity that was similar to and lower than that in the flowers.

### 3.3. Light Microscopy Analysis of Koelreuteria paniculata Aerial Parts

**Figure 2:**
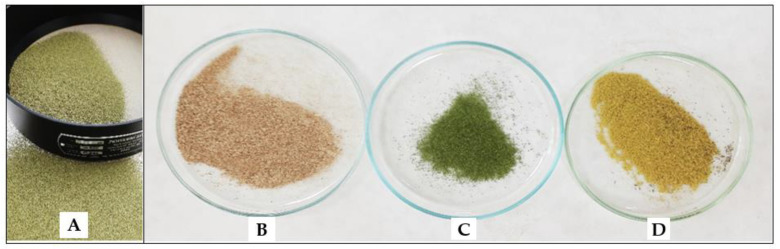
Pharmacopoeial sieve (**A**) and powdered samples of *K. panniculata*, stem bark (**B**), leaves (**A**,**C**), and flowers with flower buds (**D**).

The identification of medicinal plant substances is the first step in conducting pharmacognostic analysis, where microscopic examination plays an important role. Such type of analysis has not been reported up to date for *K. paniculata*. The objects of microscopic analysis were powdered stem bark, leaves, flowers, and flower buds (Figure 2).

**Figure 3:**
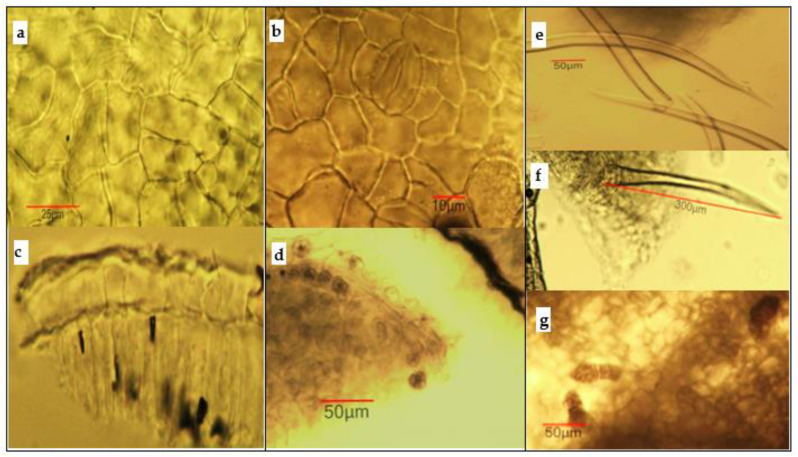
Microphotos of powdered leaf samples of *Koelreuteria paniculata*: (**a**) upper leaf epidermis in surface view; (**b**) lower leaf epidermis in surface view, with anomocytic stomata; (**c**) fragments of leaf lamina in transverse section with upper epidermis and palisade parenchyma; (**d**) oxalate druses around the vascular bundle; (**e**) isolated covering trichomes; (**f**) unicellular-covering trichome with part of the epidermis; (**g**) glandular trichomes of lower leaf epidermis.

*Microscopical examination of the powdered leaf sample*. The powder had a grassy green color and showed the following main diagnostic features (using *chloral hydrate solution*). Figure 3: fragments of the leaf epidermis in surface view consisting of straight-walled cells and anomocytic stomata; fragments of leaf lamina in the cross section containing epidermal cells and elements of chlorenchyma tissue; cluster crystals of calcium oxalate surrounding the vascular bundles, sometimes included in the parenchyma (about 13–19 μm in diameter); rare isolated conical unicellular-covering trichomes (about 400–450 μm long) and small glandular trichomes (about 50 μm long) with multicellular head and short multicellular stalk, mostly on the leaf veins of the lower surface.

**Figure 4:**
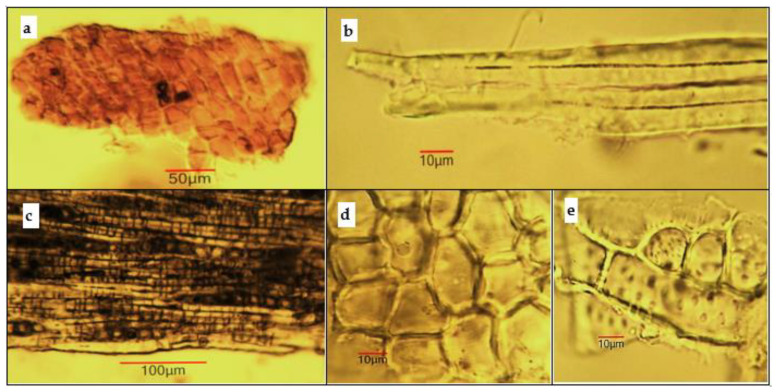
Microphotos of powdered stem bark samples of *Koelreuteria paniculata*: (**a**) cork on surface view; (**b**) isolated phloem fiber bundle; (**c**) fibers with a sheath of calcium oxalate; (**d**) parenchymal cells on surface view; (**e**) rectangular sclereids.

*Microscopical examination of powdered stem bark sample*. The powder is light brown. Examined under a microscope using *chloral hydrate solution*, the powder shows the following diagnostic features (Figure 4): fragments of cork tissue containing cells with thickened and colored red–brown cell walls; bundles of phloem fibers with very thick walls, isolated or included in phloem tissue, surrounded by a crystal sheath containing druses of calcium oxalate; fragments of parenchymal cells of the phloem; fragment of sclereids, which have thick and pitted walls.

**Figure 5:**
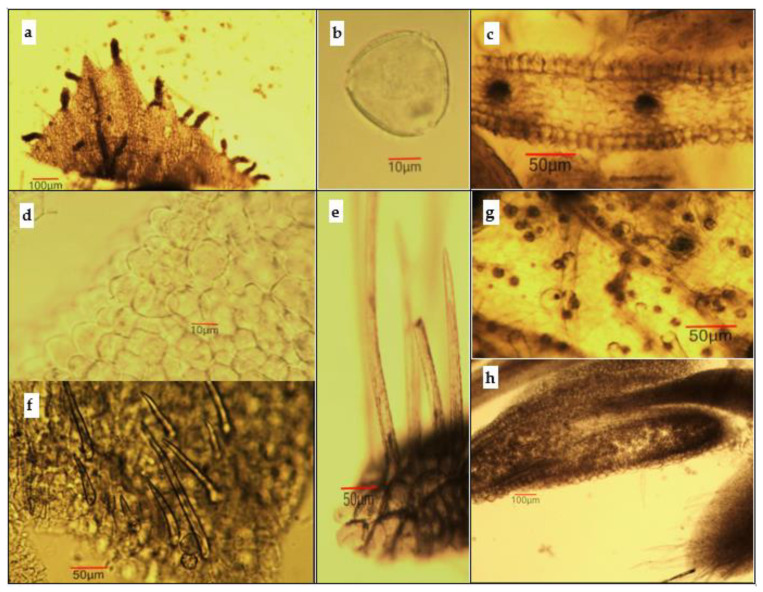
Microphotos of powdered flower and flower bud samples of *Koelreuteria paniculata:* (**a**) fragment of corolla and pollen grains; (**b**) pollen grain; (**c**) fragment of the corolla in cross section; (**d**) papillose epidermal cells of the corolla in surface view; (**e**) fragment of calyx with unicellular-covering trichomes with surface inlays; (**f**) fragment of calyx with covering trichomes; (**g**) fragment of calyx with oxalate druses; (**h**) fragment of the flower bud with anther and parts of perianthium.

*Microscopical examination of powdered flower and flower bud samples*. The powder had a yellowish color and showed the following diagnostic features (using *chloral hydrate solution*). Figure 5: triangular-ovoid or rounded pollen grains about 30 μm in diameter, which have three pores and a smooth exine; fragments of corolla containing papillose epidermal cells, anomocytic type of stomata, multiple multicellular glands (50–70 μm long) located mainly to the top of the corolla, along its edge, and covering unicellular trichomes 100–300 μm long with surface inlays; fragments of calyx with unicellular covering trichomes; some cells containing crystalline druses of calcium oxalate, located mostly around the veins; fragments of ovary and anther can also be present.

## 4. Discussion

HPLC analysis of phenolic compounds showed that the leaves extract possessed the highest amount of the identified flavonoids. Other authors [18,19] also found that leaves are a source of glycosides of quercetin and kaempferol, as well as the flavonoids—galloylepicatechin, isorhamnetin, hyperin, and 5-methoxyluteolin. Mahmoud et al. [20] also isolated from leaves of *K. paniculata* eleven phenolics, among them two new flavonol glycosides. A study of the ethanol leaf extract shows catechin content [18], while in our results, this phytocomponent was present only in the stem bark. The authors mentioned above do not provide quantitative values to compare with our results. In *Koelreuteria paniculata* flowers, Qu et al. [23] found kaempferol, luteolin, hyperin, etc., while kaempferol and hyperin were missing in our samples. The compounds in the flowers of the species studied by Qu et al. [23] were found in the ethyl acetate fraction and isolated by fractionation using various solvents and column chromatography. The reason for the difference observed in the component composition is probably due to the different methods and types of solvents used in the isolation of the compounds, as well as the different climatic and geographical conditions of the habitat. Regarding the flower buds of *K. paniculata*, there is a lack of research on their chemical composition, including the phenolic component content. There are data only for their essential oil composition, mentioned in our previous study [26], where fatty acids (linolenic acid, linoleic acid, oleic acid, palmitic acid, etc.) were of the highest concentrations.

The major flavonoids identified in this study—rutin, hesperidin, (−)-epicatechin, and quercetin—are valuable bioactive compounds that have multiple pharmacological activities. Rutin, as a natural antioxidant, is an active substance in many herbal medicines [36]. Pandey et al. [37] overviewed *in vitro* and *in vivo* studies on rutin-mediated antitumor activities. Various action mechanisms have been reported (inhibition of cell proliferation, tumor growth and metastasis, protection from carcinogenesis by enzyme modulation, and others). Its efficacy has been proven *in vivo* for various cancers (cervical cancer, leukemia, breast, prostate, liver, colon cancers, and glioblastoma). We also proved significant *in vitro* antiproliferative activity on the HT-29 cell line (human colon adenocarcinoma) for flower and leaf extracts [27]. The comparison between the amounts of flavonoids detected by HPLC-analysis and the antitumor activity found shows that the content of rutin is highest in these two plant parts (flowers and leaves) of all flavonoids, which gives us reason to assume its direct participation in the observed biological activity. The flavanone hesperidin manifests cardiovascular protection and various biological activities like anti-inflammatory, anticancer, and antifungal [8]. The authors reported that hesperidin induced apoptosis in several cancer cells like breast, ovary, prostate, and colon, and it showed hepatoprotective effects against the development of hepatocellular carcinoma. For many of the flavonoids (quercetin, rutin, catechin, epicatechin) identified in our samples, an antihypertensive effect was found [38]. Quercetin, kaempferol, and rutin also possessed *in vivo* and *in vitro* hypoglycemic effects studied through various mechanisms of action [39]. Batiha et al. [40] also reported different biological activities of quercetin and possibilities for allergy treatments, arthritis, cardiovascular diseases, etc. Quercetin inhibited *in vitro* growth of malignant tumor cells (leukemia, ovarian, breast, and colon cancers) as well [8]. Bernatova [41] reported the positive effect of (−)-epicatechin on the cardiovascular and nervous systems. This flavonoid also prevented oxidative damage and endothelial dysfunction leading to hypertension and some brain disorders. Dias et al. [8] indicated that catechin and epicatechin demonstrate anticancer, antibacterial, and antiviral activities. Various mechanisms of antimicrobial action of catechins are described by Górniak et al. [42]. In this study, catechin was found only in the stem bark, which may be related to the most effective antibacterial activity reported in our previous study [27]. Epicatechin, which was also a well-represented flavonoid in the bark, is probably also relevant to the proven antimicrobial effect of this plant extract. For the plant itself, this flavonoid possibly plays a protective role against pathogens. Ghahari et al. [21] analyzed methanol leaf extracts and identified 80 phytocomponents in three of the studied biologically active fractions, where gallic acid is the best-represented component (51.63%), followed by isobutyl gallate and benzoic acid. The active fraction (dichloromethane) against *Bacillus subtilis* analyzed by the same authors contains mainly simple phenols and their esters, of which pyrogalol is the most strongly represented (4.85%), followed by phenol, catechol, guiacol, 2,6,-dimethoxyphenol and others, which are present in significantly smaller quantities (0.46–0.11%). Flavonoids in their fractions have not been described using the GC/MS method. Chinese researchers [22], using the GC/MS method, found the availability of phenolic compounds—3,4,5,-trihydroxy-, methyl ester of benzoic acid; ethyl gallate, and pyrogallol—in leaf extracts only, but not in branches. Through the same technique and methods, extracts from other plant parts—roots [43], stem bark, and wood [44]—show the absence of phenolic components in the leaf. Other authors indicate the content of gallic acid in fresh leaves [18,19] and flowers [23] of *K. paniculata* and its derivatives (p-; m-digalloyl acid, ethyl p-trigallate, methyl m-digallate, methyl-, ethyl-gallate, and other). Our study confirmed the presence of gallic acid in leaves only, rather than in the other aerial plant parts.

Phenolic acids are widespread, and they have been documented for a number of their health-protective effects [11]. Rosmarinic acid, which was of the highest content of all 14 components we studied (over 10 mg/g DW), is a naturally occurring phenolic compound in a number of plants. Nadeem et al. [45] indicated its good therapeutic options against several diseases and significant biological effects (antibacterial, antiviral, anti-inflammatory, antioxidant, antidepressant, anticancer, antiaging activities, etc.). Rosmarinic acid shows the best antioxidant potential in a comparative evaluation of antioxidant, antimicrobial, and cytotoxic activities [46]. *P*-coumaric acid is an effective antioxidant in different *in vitro* assays [47]. The authors point out that it prevents lipid oxidation in food products and could be used to extend their shelf life and quality. In their review, Ferreira et al. [48] point out that *p*-coumaric acid is a compound that can be an effective neuroprotective, antioxidant, antineoplastic, anti-inflammatory, antimicrobial, hepato-, and nephron-protective agent. It has an inhibitory effect on human lung (A549), colon cancer cell lines (HT29-D4; HCT-15), human skin melanoma cells, ovarian cancer, breast cancer, and stomach cancer by reducing the levels of free radicals formed [48,49]. *P*-coumaric acid was found in the highest amounts in flowers and flower buds in our samples (in the range of 5–7 mg/g DW), which could explain the high antioxidant and DNA-protective effects of the extracts obtained from these two plant parts. Salicylic acid (SA), the best represented in flower buds and flowers (1.6 and 0.8 mg/g DW, respectively), is known for its anti-inflammatory and antipyretic properties, and it is important in the production of drugs such as aspirin and medical care products for skincare [50]. The highest antioxidant activity of the flower extract in this study could be explained by the highest content of protocatechuic, *p*-coumaric, and ferulic acid compared with the other plant parts, as well as by the synergistic action with the other phenolic compounds identified. Vanillic acid was among the well-represented phenolic acids in our samples (1 mg/g in leaves), which is known for its aromatic properties, but it is also referred to as an antitumor agent that inhibits proliferation and induces apoptosis in cancer cells [49]. The authors prove in vitro antioxidant capacity of plant extracts that contain vanillic acid as it reduces H_2_O_2_-induced DNA damage. Similarly, they found strong antimicrobial, anti-inflammatory, and anticancer activities of gallic acid in our study in *K. paniculata* leaves only (over 1.0 mg/g DW) and of caffeic acid, which could be seen in our samples in significantly lower quantities.

Polyphenols are the major plant compounds with antioxidant activity due to their redox properties, such as scavenging and neutralizing free radicals, quenching singlet and triplet oxygen, and decomposing peroxides [51]. As it can be seen from the literature data, antioxidant properties and DNA protective effects have been studied on methanol leaf extracts from *K. paniculata* and their fractions. Kumar et al. [15], using four methods of ABTS, DPPH, reducing power, and Superoxide anion radical scavenging assay, proved antioxidant activity, which is higher in methanol (dose-dependent) compared with its hexane fraction. The authors attribute the result to the total phenols and flavonoids available in the extract, which are missing in the fraction. The same researchers found gene-protective activity on the plasmid and genomic DNA from the thymus for both the extract and the fraction, which they associate with the presence of nonphenolic components. Other studies [13] also prove DNA-protective activity against damage caused by 4 nitroquinoline-1-oxide (4NQO), for methanol leaf extract and its fractions, with the highest one for the ethyl acetate fraction. H_2_O_2_-induced DNA damages can be effectively eliminated with the participation of leaf methanol extract and its fractions [16]. The studied carotenoid fraction of *K. paniculata* flowers showed good antioxidant activity by the ABTS assay [52]. Our data confirm the high antioxidant potential and DNA-protective effect of *K. paniculata* leaf extracts, and the manifestation of these biological activities of extracts from other aerial parts of the plant (flowers, flower buds, and bark) is reported for the first time.

## 5. Conclusions

In conclusion, the present study supposed that *Koelreuteria paniculata* extracts may be a prospective source of natural antioxidants suitable for pharmaceutical use. Fourteen phenolic compounds were identified—five flavonoids and nine phenolic acids—in the aerial plant parts studied. The HPLC analysis revealed a high content of the flavonoids rutin, hesperidin, and quercetin in the leaf extracts, as well as (−)-epicatechin in the flower bud extract. Among the identified phenolic acids, the best represented five are, in the descending order, rosmarinic, *p*-coumaric, salicylic, vanillic, and gallic acids. All *K. paniculata* extracts tested showed antioxidant and DNA-protective potential, most pronounced for the flower parts and leaves. In addition, a pharmacognostic description of the microscopy diagnostic features of the studied herbal substances has been made, which was previously lacking. The data obtained are a good basis for further research into developing herbal medicines as an alternative for the prevention and treatment of many human diseases.

## Figures and Tables

**Table 1:** Content of flavonoids and phenolic acids in extracts of *Koelreuteria paniculata* aerial parts (mg/g DW).

		Plant Aerial Parts			
No.	Compounds	Leaves	Stem Bark	Flowers	Flower Buds
	**Flavonoids**				
1	Rutin	4.23 ± 0.96 ^a^	0.03 ± 0.01 ^b,c^	0.34 ± 0.08 ^b^	0.24 ± 0.09 ^b,c^
2	Hesperidin	2.97 ± 0.42 ^a^	n.d.	0.37 ± 0.07 ^b^	0.19 ± 0.06 ^b^
3	Quercetin	2.66 ± 0.54 ^a^	0.04 ± 0.01 ^b,c^	0.42 ± 0.09 ^b^	0.24 ± 0.04 ^b,c^
4	(+)-Catechin	n.d.	0.09 ± 0.02	n.d.	n.d.
5	(−)-Epicatechin	0.38 ± 0.06 ^b,c^	0.80 ± 0.14 ^b^	0.59 ± 0.05 ^b,c^	2.69 ± 0.82 ^a^
	**Phenolic acids**				
6	Gallic	1.02 ± 0.22	n.d.	n.d.	n.d.
7	Protocatehuic	0.30 ± 0.10 ^c^	traces	0.75 ± 0.10 ^a^	0.53 ± 0.06 ^b^
8	Vanillic	1.04 ± 0.08 ^a^	0.19 ± 0.04 ^b,c^	0.24 ± 0.04 ^b^	0.14 ± 0.05 ^b,c^
9	Caffeic	0.06 ± 0.02 ^n.s.^	0.11 ± 0.03 ^n.s.^	0.10 ± 0.02 ^n.s.^	0.14 ± 0.08 ^n.s.^
10	Syringic	0.13 ± 0.07 ^a,b,c^	0.07 ± 0.02 ^c^	0.23 ± 0.08 ^a,b^	0.24 ± 0.06 ^a^
11	*p*-Coumaric	0.26 ± 0.06 ^c^	0.05 ± 0.01 ^c^	6.97 ±1.04 ^a^	4.97 ± 0.97 ^a,b^
12	Ferulic	0.07 ± 0.02 ^b^	n.d.	0.13 ± 0.04 ^b^	0.94 ± 0.2 ^a^
13	Salicylic	0.39 ± 0.04 ^b,c^	0.10 ± 0.03 ^b,c^	0.77 ± 0.17 ^a,b^	1.64 ± 0.65 ^a^
14	Rosmarinic	10.34 ± 1.80 ^a^	0.22 ± 0.08 ^c^	3.00 ± 0.38 ^b^	2.62 ± 0.93 ^b,c^

n.d.—not detected; The samples were analyzed in triplicate, and results were expressed in mean ± standard deviation (SD). Different superscript letters indicate significant differences according to Tukey’s test (*p* < 0.01). ^n.s.^—not significant.

**Table 2:** *In vitro* antioxidant activities of extracts from different *Koelreuteria paniculata* plant parts.

Sample ^3^	DPPH-Assay ^2^, mmol TE/g DW ^1^	ABTS-Assay, mmol TE/g DW	FRAP-Assay, mmol TE/g DW	CUPRAC-Assay, mmol TE/g DW
LE	751.27 ± 1.27 ^c^	645.88 ± 1.83 ^c^	1838.92 ± 2.42 ^c^	576.68 ± 2.58 ^d^
SBE	278.39 ± 1.44 ^d^	342.55 ± 0.98 ^d^	637.62 ± 3.16 ^d^	846.16 ± 2.17 ^b^
FE	1133.47 ± 1.97 ^a^	1437.49 ± 0.76 ^a^	4308.02 ± 2.84 ^a^	1748.50 ± 2.69 ^a^
FBE	904.12 ± 1.75 ^b^	686.68 ± 1.45 ^b^	2464.10 ± 2.93 ^b^	731.81 ± 1.88 ^c^

^1^ mmolTE/g DW—mmol Trolox equivalent (Trolox: 6-hydroxy-2,5,7,8-tetramethylchroman-2-carboxylic acid) per gram of dry weight; ^2^ DPPH—2,2-diphenil-1-picrylhydrazyl; ABTS:—2,2′-Azino-bis (3-ethylbenzothiazoline-6-sulfonic acid) FRAP—Ferric reducing antioxidant power; CUPRAC—Cupric reducing antioxidant capacity; ^3^ LE—leaf extract; SBE—stem bark extract; FE—flower extract; FBE—flower buds extract; The samples were analyzed in triplicate, and results were expressed in mean ± standard deviation (SD). Different superscript letters indicate significant differences according to Tukey’s test (*p* < 0.01).

## Supplementary Materials

The following supporting information can be downloaded at: https://www.mdpi.com/article/10.3390/antiox11061154/s1, Figure S1: chromatographic profile of the phenolic acids and flavonoids at 280 nm—*K. paniculata* leaf extract (a), and standard mixture (b); Figure S2: chromatographic profile of the phenolic acids and flavonoids at 360 nm—*K. paniculata* leaf extract (a), and standard mixture (b).

## References

[1] Kumar S., Pandey A.K. (2013). Chemistry and Biological Activities of Flavonoids: An Overview. Sci. World J..

[2] Venkatachalapathy D., Shivamallu C., Prasad S.K., Thangaraj Saradha G., Rudrapathy P., Amachawadi R.G., Patil S.S., Syed A., Elgorban A.M., Bahkali A.H. (2021). Assessment of Chemopreventive Potential of the Plant Extracts against Liver Cancer Using HepG2 Cell Line. Molecules.

[3] Halliwell B., Adhikary A., Dingfelder M., Dizdaroglu M. (2021). Hydroxyl Radical Is a Significant Player in Oxidative DNA Damage In Vivo. Chem. Soc. Rev..

[4] Kumar M., Kumar S., Kaur S. (2012). Role of ROS and COX-2/INOS Inhibition in Cancer Chemoprevention: A Review. Phytochem. Rev..

[5] Patel K., Kumar V., Rahman M., Verma A., Patel D.K. (2018). New Insights into the Medicinal Importance, Physiological Functions and Bioanalytical Aspects of an Important Bioactive Compound of Foods ‘Hyperin’: Health Benefits of the Past, the Present, the Future. Beni-Suef Univ. J. Basic Appl. Sci..

[6] Singh R.L., Sapna Sharma S.S., Pankaj Singh P.S., Prakash D., Sharma G. (2014). Antioxidants: Their Health Benefits and Plant Sources. Phytochemicals of Nutraceutical Importance.

[7] Karak P. (2019). Biological activities of flavonoids: An overview. Int. J. Pharm Sci. Res..

[8] Dias M.C., Pinto D.C.G.A., Silva A.M.S. (2021). Plant Flavonoids: Chemical Characteristics and Biological Activity. Molecules.

[9] Tungmunnithum D., Thongboonyou A., Pholboon A., Yangsabai A. (2018). Flavonoids and Other Phenolic Compounds from Medicinal Plants for Pharmaceutical and Medical Aspects: An Overview. Medicines.

[10] Kiokias S., Proestos C., Oreopoulou V. (2020). Phenolic Acids of Plant Origin—A Review on Their Antioxidant Activity In Vitro (O/W Emulsion Systems) Along with Their in Vivo Health Biochemical Properties. Foods.

[11] Kumar N., Goel N. (2019). Phenolic Acids: Natural Versatile Molecules with Promising Therapeutic Applications. Biotechnol. Rep..

[12] Ljubojević M., Tomić M., Simikić M., Savin L., Narandžić T., Pušić M., Grubač M., Vejnović S., Marinković M. (2021). *Koelreuteria paniculata* Invasiveness, Yielding Capacity and Harvest Date Influence on Biodiesel Feedstock Properties. J. Environ. Manag..

[13] Kumar M. (2011). Golden Rain Tree Leaf Extracts as Potential Inhibitor of Lipid Peroxidation and 4-Nitroquinoline-1-Oxide (4-NQO)-Induced DNA Damage. Afr. J. Biotechnol..

[14] Kumar M. (2011). Investigations on DNA Protective and Antioxidant Potential of Chloroform and Ethyl Acetate Fractions of *Koelreuteria paniculata* Laxm. Afr. J. Pharm. Pharmacol..

[15] Kumar M., Chandel M., Kumar S., Kaur S. (2012). Studies on the Antioxidant/Genoprotective Activity of Extracts of *Koelreuteria paniculata* Laxm. Am. J. Biomed. Sci..

[16] Kumar M., Chandel M., Sharma N., Kumar S., Kaur S. (2012). Efficacy of Golden Rain Tree against Free Radicals and H2O2-Induced Damage to PUC18/Calf Thymus DNA. Asian Pac. J. Trop. Biomed..

[17] Chunyi T., Wen D., Zhongsong G. (2005). The Total Flavon Extraction from Fruits, Branchs, Leaves of the *Koelreuteria paniculata* Laxm and It,s Content Determination. Chin. Agric. Sci. Bull..

[18] Lin W.-H., Deng Z.-W., Lei H.-M., Fu H.-Z., Li J. (2002). Polyphenolic Compounds from the Leaves of *Koelreuteria paniculata* Laxm. J. Asian Nat. Prod. Res..

[19] Mostafa A.E., El-Hela A.A., Mohammad A.-E.I., Cutler S.J., Ross S.A. (2016). New Triterpenoidal Saponins from *Koelreuteria paniculata*. Phytochem. Lett..

[20] Mahmoud I., Moharram F.A., Marzouk M.S., Soliman H.S.M., El-Dib R.A. (2001). ChemInform Abstract: Two New Flavonol Glycosides from Leaves of *Koelreuteria paniculata*. Die Pharm..

[21] Ghahari S., Alinezhad H., Nematzadeh G.A., Ghahari S. (2015). Phytochemical Screening and Antimicrobial Activities of the Constituents Isolated from *Koelreuteria paniculata* Leaves. Nat. Prod. Res..

[22] Wang Y., Zheng D., Zhao Y., Wang T., Yang Y., Aqeel M., Peng W. (2018). Active Components in Branches and Leaves of *Koelreuteria paniculata*. Caribb. J. Sci..

[23] Qu Q.-H., Zhang L., Bao H., Zhang J.-H., You X.-J., Wang J.-X. (2011). Chemical Constituents of Flavonoids from Flowers of *Koelreuteria paniculata*. J. Chin. Med. Mater..

[24] Yang X., Lei H., Fu H., Lin W. (2000). Study on the flavonoids from the seeds of *Koelreuteria paniculata* Laxm. Acta Pharm. Sin..

[25] Sutiashvili M.G., Alaniya M.D., Mshvildadze V.D., Skhirtladze A.V., Pichette A., Lavoie S. (2013). Flavonoid and Cycloartane Glycosides from Seeds of *Koelreuteria paniculata*. Chem. Nat. Compd..

[26] Andonova T., Dimitrova-Dyulgerova I., Slavov I., Muhovski Y., Stoyanova A. (2020). A Comparative Study of *Koelreuteria paniculata* Laxm. Aerial Parts Essential Oil Composition. J. Essent. Oil Bear. Plants.

[27] Andonova T., Muhovski Y., Fidan H., Slavov I., Stoyanova A., Dimitrova-Dyulgerova I. (2021). Chemical Compounds, Antitumor and Antimicrobial Activities of Dry Ethanol Extracts from *Koelreuteria paniculata* Laxm. Plants.

[28] Krasteva G. (2022). Effect of Basal Medium on Growth and Polyphenols Accumulation by Gardenia Jasminoides Ellis Cell Suspension. BIO Web Conf..

[29] Kivrak İ., Duru M.E., Öztürk M., Mercan N., Harmandar M., Topçu G. (2009). Antioxidant, Anticholinesterase and Antimicrobial Constituents from the Essential Oil and Ethanol Extract of *Salvia Potentillifolia*. Food Chem..

[30] Ivanov I.G., Vrancheva R.Z., Marchev A.S., Petkova N.T., Aneva Y., Denev P.P., Georgiev V.G., Pavlov A.I. (2014). Antioxidant Activities and Phenolic Compounds in Bulgarian Fumaria Species. Int. J. Curr. Microbiol. App. Sci..

[31] Thaipong K., Boonprakob U., Crosby K., Cisneros-Zevallos L., Byrne D.H. (2006). Comparison of ABTS, DPPH, FRAP, and ORAC Assays for Estimating Antioxidant Activity from Guava Fruit Extracts. J. Food Compos. Anal..

[32] Benzie I.F.F., Strain J.J. (1999). [2] Ferric Reducing/Antioxidant Power Assay: Direct Measure of Total Antioxidant Activity of Biological Fluids and Modified Version for Simultaneous Measurement of Total Antioxidant Power and Ascorbic Acid Concentration. Methods in Enzymology.

[33] Apak R., Güçlü K., Özyürek M., Karademir S.E., Erçağ E. (2006). The Cupric Ion Reducing Antioxidant Capacity and Polyphenolic Content of Some Herbal Teas. Int. J. Food Sci. Nutr..

[34] Rajiv C., Roy S.S., Tamreihao K., Kshetri P., Singh T.S., Devi H.S., Sharma S.K., Ansari M.A., Devi E.D., Devi A.K. (2021). Anticarcinogenic and Antioxidant Action of an Edible Aquatic Flora *Jussiaea repens* L. Using In Vitro Bioassays and In Vivo Zebrafish Model. Molecules.

[35] Vasavada N. Online Web Statistical Calculators. http://astatsa.com/.

[36] Mao Y.-J., Feng Y.-L., Wang M.-J., Lyu Z.-Y., Zhai G.-Y. (2021). Research Progress on Rutin Derivatives. China J. Chin. Mater. Med..

[37] Pandey P., Khan F., Qari H.A., Oves M. (2021). Rutin (Bioflavonoid) as Cell Signaling Pathway Modulator: Prospects in Treatment and Chemoprevention. Pharmaceuticals.

[38] Cao Y., Xie L., Liu K., Liang Y., Dai X., Wang X., Lu J., Zhang X., Li X. (2021). The Antihypertensive Potential of Flavonoids from Chinese Herbal Medicine: A Review. Pharmacol. Res..

[39] Yen F.S., Qin C.S., Xuan S.T.S., Ying P.J., Le H.Y., Darmarajan T., Gunasekaran B., Salvamani S. (2021). Hypoglycemic Effects of Plant Flavonoids: A Review. Evid. Based Complement. Altern. Med..

[40] Batiha G.E.-S., Beshbishy A.M., Ikram M., Mulla Z.S., El-Hack M.E.A., Taha A.E., Algammal A.M., Elewa Y.H.A. (2020). The Pharmacological Activity, Biochemical Properties, and Pharmacokinetics of the Major Natural Polyphenolic Flavonoid: Quercetin. Foods.

[41] Bernatova I. (2018). Biological Activities of (−)-Epicatechin and (−)-Epicatechin-Containing Foods: Focus on Cardiovascular and Neuropsychological Health. Biotechnol. Adv..

[42] Górniak I., Bartoszewski R., Króliczewski J. (2019). Comprehensive Review of Antimicrobial Activities of Plant Flavonoids. Phytochem. Rev..

[43] Wang Y., Liu Q., Zheng D., Zhao Y., Wang T., Yan S., Gu H. (2020). Active Constituents of *Koelreuteria paniculata* Root. Therm. Sci.

[44] Yang Y., Zheng D., Zhao Y., Wang T., Yang Y., Peng W. (2017). Analysis of components in *Koelreuteria paniculata*. Caribb. J. Sci..

[45] Nadeem M., Imran M., Aslam Gondal T., Imran A., Shahbaz M., Muhammad Amir R., Wasim Sajid M., Batool Qaisrani T., Atif M., Hussain G. (2019). Therapeutic Potential of Rosmarinic Acid: A Comprehensive Review. Appl. Sci..

[46] Godlewska-Żyłkiewicz B., Świsłocka R., Kalinowska M., Golonko A., Świderski G., Arciszewska Ż., Nalewajko-Sieliwoniuk E., Naumowicz M., Lewandowski W. (2020). Biologically Active Compounds of Plants: Structure-Related Antioxidant, Microbiological and Cytotoxic Activity of Selected Carboxylic Acids. Materials.

[47] Kiliç I., Yeşiloğlu Y. (2013). Spectroscopic Studies on the Antioxidant Activity of *P*-Coumaric Acid. Spectrochim. Acta. Part A.

[48] Ferreira P.S., Victorelli F.D., Fonseca-Santos B., Chorilli M. (2019). A Review of Analytical Methods for *p* -Coumaric Acid in Plant-Based Products, Beverages, and Biological Matrices. Crit. Rev. Anal. Chem..

[49] Abotaleb M., Liskova A., Kubatka P., Büsselberg D. (2020). Therapeutic Potential of Plant Phenolic Acids in the Treatment of Cancer. Biomolecules.

[50] Sambyal K., Singh R.V. (2021). Production of Salicylic Acid; a Potent Pharmaceutically Active Agent and Its Future Prospects. Crit. Rev. Biotechnol..

[51] Calderon-Montano M.J., Burgos-Moron E., Perez-Guerrero C., Lopez-Lazaro M. (2011). A Review on the Dietary Flavonoid Kaempferol. Mini Rev. Med. Chem..

[52] Zhelev I., Georgiev K., Dimitrova-Dyulgerova I. (2016). In-vitro antioxidant and antineoplastic activities of carotenoids from flowers of *Koelreuteria paniculata*. World J. Pharm. Res..

