# Supplementary material for: Antioxidant and DNA-Protective Potentials, Main Phenolic Compounds, and Microscopic Features of Koelreuteria paniculata Aerial Parts
Source: Antioxidants (Basel). 2022 Jun 13;11(6):1154. doi: 10.3390/antiox11061154 (PMC9219871; doi:10.3390/antiox11061154)
Supplement: Supplementary file 1 [file antioxidants-11-01154-s001.zip › antioxidants-1741435-supplementary.pdf]

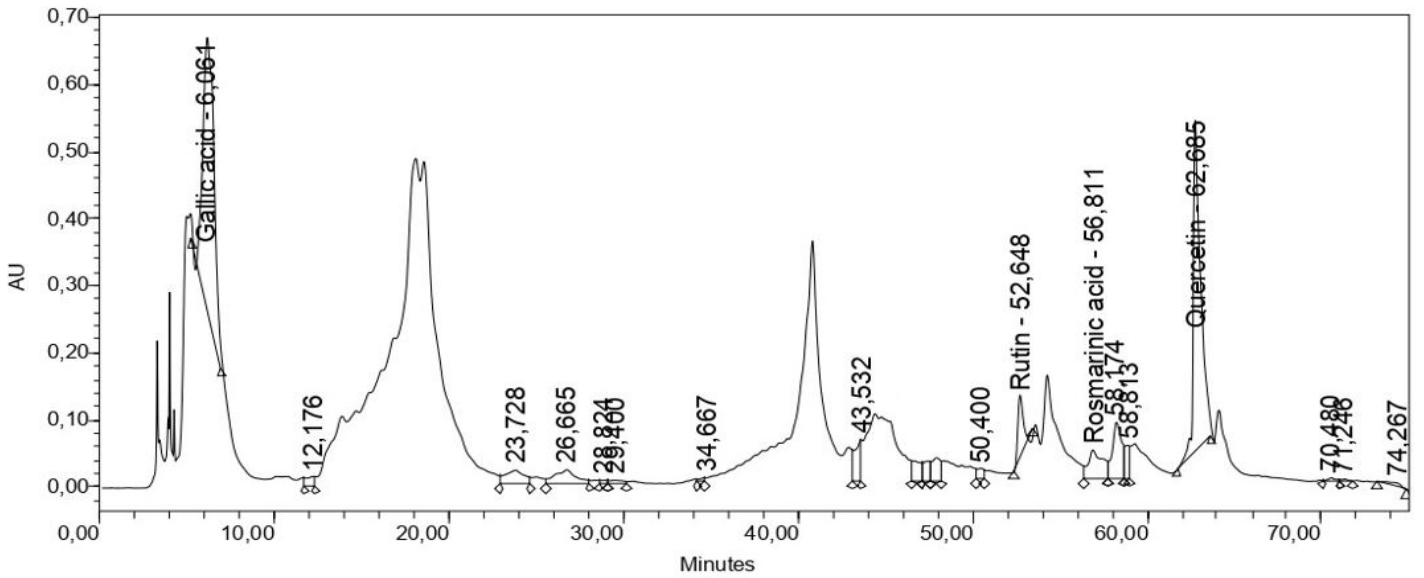

(a)

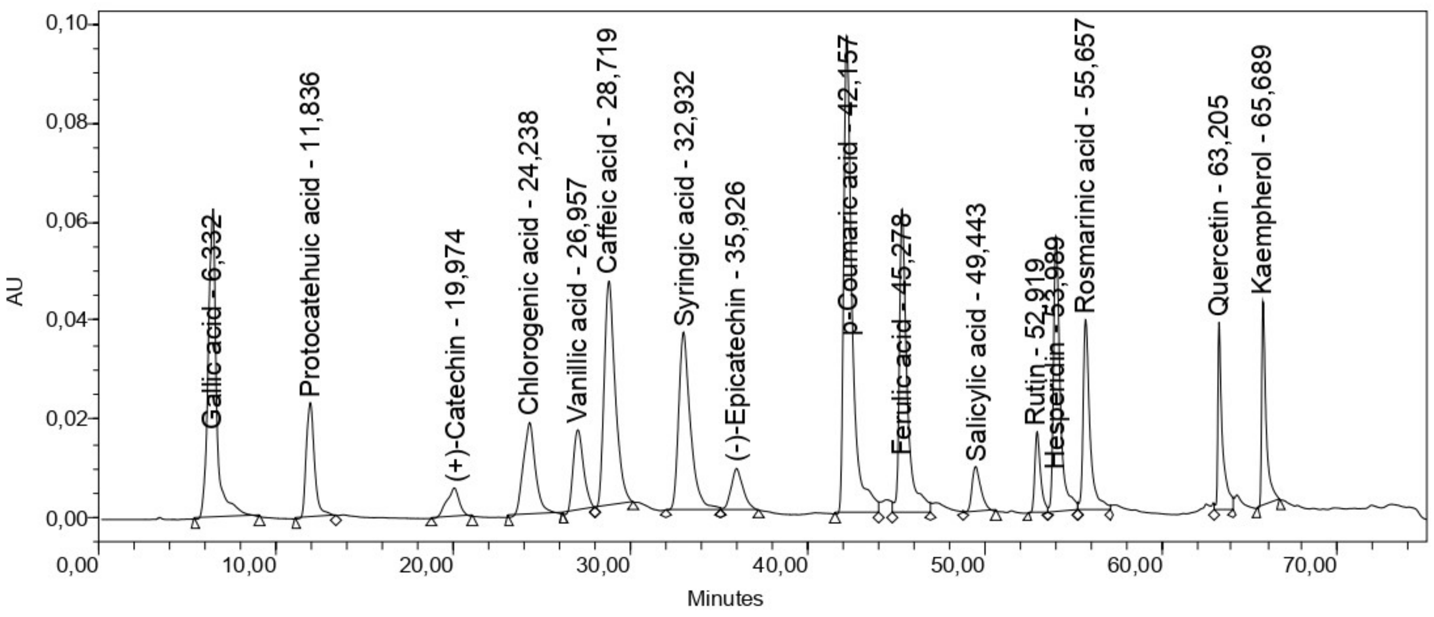

(b)

Figure S1. Chromatographic profile of the phenolic acids and flavonoids at 280 nm- *K. paniculata* leaf extract (a), and standard mixture (b).

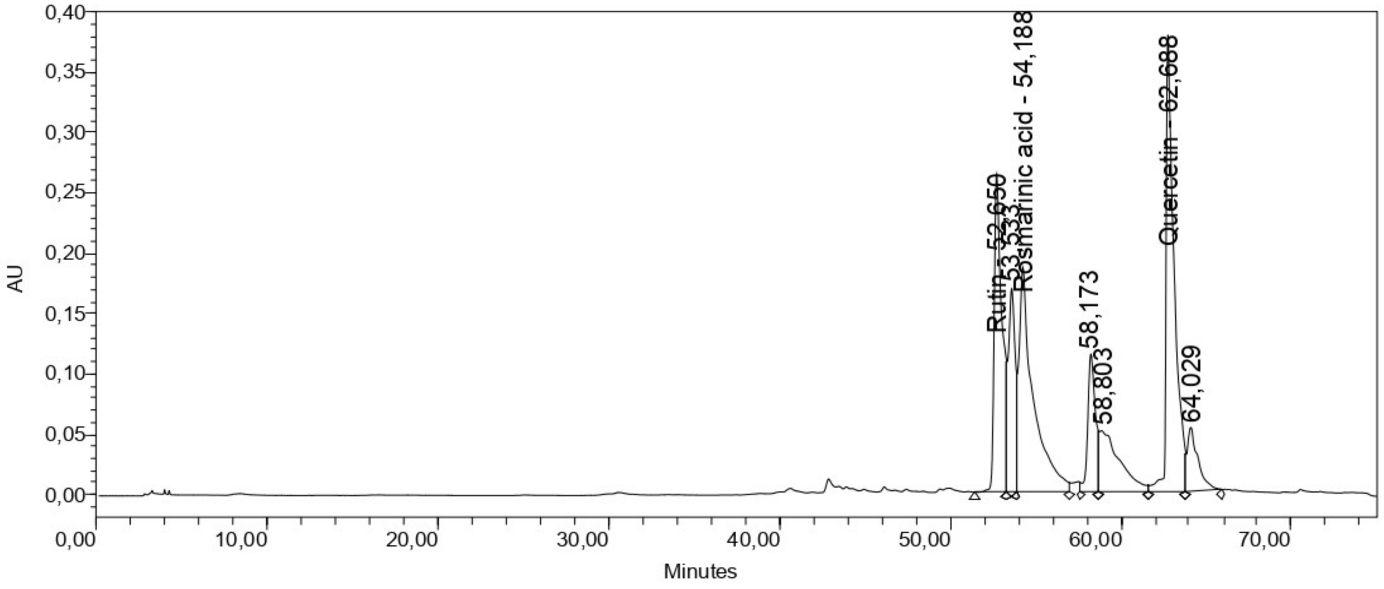

(a)

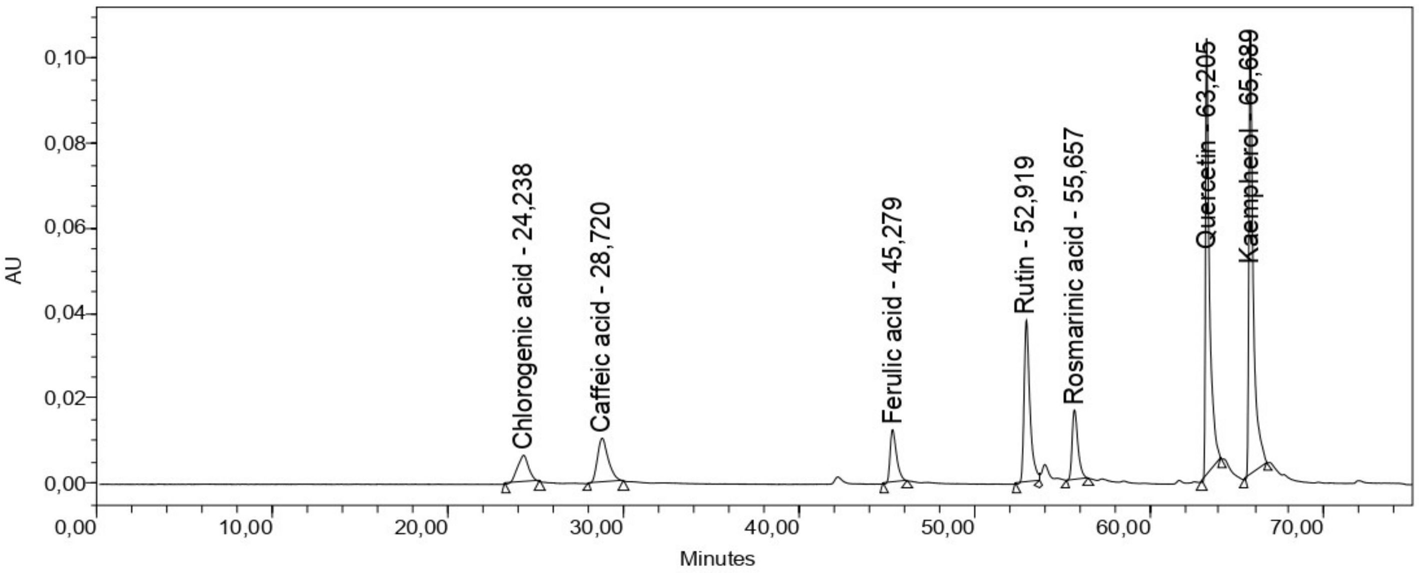

(b)

Figure S2. Chromatographic profile of the phenolic acids and flavonoids at 360 nm - *K. paniculata* leaf extract (a), and standard mixture (b).
